# Marital Satisfaction and Its Influencing Factors in Fertile and Infertile Women 

**Published:** 2016-09

**Authors:** Mohammad Amiri, Zakieh Sadeqi, Mohammad Hassan Hoseinpoor, Ahmad Khosravi

**Affiliations:** 1 Department of Public Health, School of Public Health, Shahroud University of Medical Sciences. Shahroud, Iran; 2 Department of Chemistry, Analytical Chemistry, Shahroud, Iran; 3 Department of Counseling, Non-Benefit University of Shahroud, Shahroud, Iran; 4 Center for Health Related Social and Behavioral Sciences Research, Shahroud University of Medical Sciences, Shahroud, Iran

**Keywords:** Marital Satisfaction, Sexuality, Infertility, Marital Communication, Fertility, Conflict Resolution, Idealistic Distortion

## Abstract

**Objective:** To determine marital satisfaction and its influencing factors among fertile and infertile women in Shahroud.

**Materials and methods:** In this comparative study, 1528 participants (511 infertile and1017 fertile women) were evaluated using Enrich Marital Satisfaction Scale. Data were analyzed using chi-square and t-test.

**Results:** A total of 1402 participants (78.7%) had high marital satisfaction. The results show that no significant differences exist between marital satisfaction, marital communication, conflict resolution and idealistic distortion in fertile and infertile women. However, a significant difference was observed between marital satisfaction, and job, spouse’s job and income in fertile and infertile groups, but the place of residence, education, spouse's education and fertility status showed no significant difference.

**Conclusion:** Results showed that infertility does not reduce marital satisfaction. Since marital satisfaction is moderate in both groups, sex education for people bound to marry and sexual counseling for couples can lead to improved sexual satisfaction.

## Introduction

One of the main reasons for the marriage of a man and a woman is waiting for the birth of a child in their shared life (1). Infertility is a problem and one of the bitterest experiences of life. Despite the progress in regeneration and auxiliary equipment and techniques which help infertile couples to manage and reduce fertility problems, almost 80 million people in the world are experiencing infertility in their lives (2). Studies show that fertility rate has increased by 50% since the earliest reports (3).

Infertility has been defined as the inability to conceive after one year of regular sexual intercourse without using a contraceptive method (-6). Countless numbers of couples are affected by infertility so that a number of studies have reported the incidence to be one pair out of 10 (5, 7, 8). The results of a study done in 2007 on 172,413 women in 25 countries reported the infertility rates in developed countries between 3.5 to 16.7 percent and in developing countries 6.9 to 9.3 percent (9-11). The results of other studies reported the prevalence of infertility in Turkey 10% (12), India 15% (13, 14) and America (10%) (15). Infertility exerts negative effects on physical and mental health and can lead to marital problems (16).

The results of some studies suggest a negative effect of infertility on marital satisfaction, while others suggest its positive effect on marital satisfaction (17, 18). Marital satisfaction is an important factor affecting family health and it is one of the indicators of life satisfaction (19), which can influence life satisfaction, job satisfaction, satisfaction with income, success and mental health and marital dissatisfaction can impair social relationships, decline cultural values and increase social deviations among the couples (20). The results of a study in Mashhad (north east of Iran) showed that only 33.6% of the people in the study had high marital satisfaction (17). In another study, the mean score of marital satisfaction in infertile women was less than that of fertile women (20). The results of studies in Tehran showed that significant differences exist in marital satisfaction in fertile and infertile women so that marital satisfaction in infertile participants was less than that in fertile ones (3, 21). A study in Turkey showed the negative impact of infertility on marital satisfaction (22). Given the importance of this topic, the current study was conducted to compare marital satisfaction in fertile and infertile women and determine the factors affecting it.

## Materials and methods

In this comparative study, to measure marital satisfaction in fertile and infertile women, Enrich Scale was used  (23, 24). In this study, 511 married infertile women aged 15-49 years old were randomly selected and studied. To compare their marital satisfaction with fertile women, 1058 such married women aged 15-49 years old who had the experience of at least one pregnancy were randomly selected and questionnaires were completed for them.

Enrich marital satisfaction scale has 4 subscales and 35 items the reliability of the subscales were found to be 0.86 for marital satisfaction, 0.80 for Communications, 0.84 for conflict resolution and 0.83 for idealistic distortion  (23). Marital Satisfaction Scale measures people’s satisfaction and compliance with the 10 aspects of the marital relationship including issues of personality, marital communication, conflict resolution, financial management, leisure activities, sex, marriage, children, relatives and friends, the role of egalitarianism and religious orientation. High scores indicate high marital satisfaction. The subscale of idealistic distortion measures the tendency of the couples based on their responses to questions on acceptable social behaviors. High scores indicate unrealistic expectation in marital relationships. The subscale of communication includes emotions, attitudes, and beliefs of the person about the degree and methods of marital relationships. The high score reflects the knowledge and consent of the couple of the level and type of their relationship, and low scores indicate a lack of satisfaction with the relationship. And the subscale of conflict resolution measures attitudes, feelings and beliefs of the partner in the creation or resolution of the conflicts also assesses the ways the couple end debates. The high score reflects realistic attitudes about the conflict in marital relationships, and low scores indicate dissatisfaction with the way conflicts are resolved. The scale includes five-point Likert type items ranging from "strongly disagree", "disagree", "neither agree nor disagree", "agree", "strongly agree", which are assigned scores from 1 to 5. Items 3-5-6-7-10-13-14-18-19-21-22-23-26-27-28-29-32-33-34 are reverse scored. In other words, in these items 1 is assigned to strongly agree and 5 is assigned to strongly disagree. The scale consists of 4 separate scores which are calculated for each subscale. The raw score is converted to a percentage. Items 1-5-9-13-17-21-24-27-30-35 belong to marital satisfaction, items 2-6-10-14-18-22-25-28-31-34 belong to communications, items 3-7-11-15-19-23-26-29-32-33 belong to conflict resolution and items 4-8-12-16-20 belong to idealistic distortion. 

This study was reviewed and approved by Shahroud University of Medical Sciences Ethical Review Board (Code Number: 9103). The collected data were entered into SPSS software and analyzed using t-test and chi-square test. Also the relation between socio-demographic variables and mean percentage score of Enrich martial satisfaction sub-scales were analyzed using a multiple regression model. In all models age was added as a covariate variable. The significance level for all tests is 0.05.

## Results

In this study, 511 women with fertility problem, (364 (71.2%) participants had primary infertility, 147 (28.8%) participants had secondary infertility) were compared with 1017 fertile women. The majority of women (1302 (85.2%)) were housewives and others were working. A total of 1402 (78.7%) participants had high marital satisfaction. The mean percentage score of marital satisfaction in fertile was 71.2 ± 12.4 and in infertile women it was 70.1 ± 12.3. Also comparison between fertile and infertile in terms of mean percentage scores of marital communication (64.3 ±12.9 vs. 65.2 ± 13.1), conflict resolution (61.0 ±10.49 vs. 61.2 ± 10.1) and idealistic distortion (70.55 ± 15.72 vs. 70.28 ± 14.89) showed no significant differences between two groups. Marital satisfaction mean percentage scores in both fertile and infertile women were above 70%, which indicate high satisfaction in all aspects of the marital relationship is in both groups. The mean percentage scores of idealistic distortion were also above 70% in the two groups of fertile and infertile women, which represents an unrealistic relationship with the partner and delaying or denying the problem in marital relationship. The mean percentage scores of the two groups in resolving the conflict were over 60%, which indicates a positive feeling in effective resolutions of debates through dialog. The mean percentage score of marital communications in infertile couples was 64.3%, which suggests a good feeling along with moderate concern about marital relations. But in fertile women it shows positive feeling with little concern about the marital relationship. In general, in none of the above-mentioned dimensions a significant difference was observed between fertile and infertile women (p > 0.05).

The relationship between socio-demographic variables and fertility status in terms of marital satisfaction subscales classification were presented in tables 1-4. The results of tables 1-4 showed no significant differences between marital satisfaction, marital communication, conflict resolution and idealistic distortion of fertile and infertile women (p > 0.05). The results of table 1 showed in univariate analysis, the job, spouse's job and family income were the main variables related to marital satisfaction levels. In a multiple regression model with mean percentage score of marital satisfaction as a dependent variable, in addition to above mentioned variables, education level of women and her husband were significant. 

**Table 1:** the relation between socio-demographic variables and marital satisfaction levels

**Variable**		**Marital Satisfaction**				**χ** ^2^	**p Value**
		**Low** **n (%)**	**Average** **n (%)**	**High** **n (%)**	**Very High** **n (%)**		
Place of Residence	Urban	1(0.2)	153 (23.7)	353 (54.7)	138 (21.4)	6.57	0.09
	Rural	4 (0.5)	168 (19)	527 (59.7)	184 (20.8)		
Job	Employed	1 (0.4)	63 (27.9)	122 (54)	40 (17.7)	8.10	0.04
	Housewife	4 (0.3)	258 (19.8)	758 (58.2)	282 (21.7)		
Education	Illiterate	0 (0)	11 (28.9)	23 (60.5)	4 (10.5)	9.76	0.370
	Below diploma	2 (0.3)	146 (20.5)	427 (59.9)	138 (19.4)		
	High school Diploma	1 (0.2)	105 (21.7)	271 (56)	107 (22.1)		
	Higher Education	2 (0.7)	59 (20.1)	159 (54.3)	73 (24.9)		
Spouse’s Education	Illiterate	0 (0)	0 (0)	4 (80)	1 (20)	8.01	0.533
	Below diploma	3 (0.4)	163 (22)	430 (58.1)	144 (19.5)		
	High school Diploma	0 (0)	33 (19)	109 (62.6)	32 (18.4)		
	Higher Education	2 (0.3)	125 (20.5)	337 (55.3)	145 (23.8)		
Spouse’s Job	Unemployed	1 (2.4)	18 (42.9)	21 (50)	2 (4.8)	21.27	0.001
	Employed	4 (0.3)	303 (20.4)	859 (57.8)	320 (21.5)		
Income	Less than 100$	1 (0.2)	84 (20.3)	247 (59.7)	82 (19.8)	13.86	0.031
	100-300$	3 (0.3)	163 (18.9)	502 (58.3)	193 (22.4)		
	More than 300$	1 (0.4)	74 (29.2)	131 (51.8)	47 (18.6)		
Fertility Status	Fertile	2 (0.2)	207 (20.4)	584 (57.4)	224 (22.0)	3.53	0.317
	Infertile	3 (0.6)	114 (22.3)	296 (57.9)	98 (19.2)		

**Table 2:** the relation between socio-demographic variables and marital communication levels

**Variable**		**Marital Communication**				**χ** ^2^	**P Value**
		**Low** **n (%)**	**Average** **n (%)**	**High** **n (%)**	**Very High** **n (%)**		
Place of Residence	Urban	4 (0.6)	245 (38)	310 (48.1)	86 (13.3)	4.86	0.182
	Rural	11 (1.2)	364 (41.2)	414 (46.9)	94 (10.6)		
Job	Employed	1 (0.4)	89 (39.4)	109 (48.2)	2 (11.9)	0.85	0.84
	Housewife	14 (1.1)	520 (39.9)	615 (47.2)	15 (11.8)		
Education	Illiterate	0 (0)	16 (42.1)	19 (50)	3 (7.9)	15.31	0.08
	Below diploma	8 (1.1)	301 (42.2)	338 (47.4)	6 (9.3)		
	High school Diploma	5 (1)	194 (40.1)	221 (45.7)	64 (13.2)		
	Higher Education	2 (0.7)	98 (33.4)	146 (49.8)	47 (16)		
Spouse’s Education	Illiterate	0 (0)	0 (0)	3 (60)	2 (40)	11.55	0.240
	Below diploma	10 (1.4)	304 (41.1)	346 (46.8)	80 (10.8)		
	High school Diploma	1(0.6)	75(43.1)	81(46.6)	17(9.8)		
	Higher Education	4(0.7)	230(37.8)	294(48.3)	81(13.3)		
Spouse’s Job	Unemployed	1 (2.4)	20 (47.6)	19 (54.2)	2 (4.8)	3.36	0.339
	Employed	14 (0.9)	589 (39.6)	705 (47.4)	178 (12)		
Income	Less than 100$	4 (1.0)	170 (41.1)	200 (48.3)	40 (9.7)	3.71	0.716
	100-300$	9 (1)	333 (38.7)	412 (47.9)	107 (12.4)		
	More than 300$	2 (0.8)	106 (41.9)	112 (44.3)	33 (13)		
Fertility Status	Fertile	11 (1.1)	379 (39)	485 (47.7)	124 (12.2)	1.32	0.72
	Infertile	4 (0.8)	212 (41.5)	239 (46.8)	56 (11)		

The results of table 2 showed there is not a significant relation between socio-demographic variables and categorical levels of marital communication. But in multiple model, the occupied women and increasing in spouse's education level were associated with increasing in mean percentage score of marital communication. There is significant relation between residency location, education and family income with conflict resolution levels (table 3) and in multiple regression, women with high level of education and family income had higher mean percentage scores. Table 4 indicated spouse's job and family income were associated with idealistic distortion classification levels. In the regression model, in addition to spouse's job and family income, the variables of education and spouse's education were significant.

**Table 3:** the relation between socio-demographic variables and conflict resolution levels

**Variable**		**Conflict Resolution**				**χ** ^2^	**P Value**
		**Low** **n (%)**	**Average** **n (%)**	**High** **n (%)**	**Very High** **n (%)**		
Place of Residence	Urban	3 (0.5)	289 (44.8)	319 (49.5)	34 (5.3)	23.55	0.001
	Rural	2 (0.2)	479 (54.2)	386 (43.7)	16 (1.8)		
Job	Employed	1 (0.4)	104 (46)	111 (49.1)	10 (4.4)	2.64	0.45
	Housewife	4 (0.3)	664 (51)	594 (45.6)	40 (3.1)		
Education	Illiterate	0 (0)	25 (65.8)	13 (34.2)	0 (0)	58.53	0.001
	Below diploma	4 (0.6)	380 (53.3)	314 (44)	15 (2.1)		
	High school Diploma	0 (0)	243 (50.2)	234 (48.3)	7 (1.4)		
	Higher Education	1 (0.3)	120 (41)	144 (49.1)	28 (9.6)		
Spouse’s Education	Illiterate	0 (0)	2 (40)	3 (60)	0 (0)	10.93	0.280
	Below diploma	3 (04)	394 (53.2)	324 (43.8)	19 (2.6)		
	High school Diploma	1 (0.6)	90 (51.7)	79 (45.4)	4 (2.3)		
	Higher Education	1 (0.2)	282 (46.3)	299 (49.1)	27 (4.4)		
Spouse’s Job	Unemployed	0 (0)	23 (54.8)	19 (45.2)	0 (0)	1.74	0.629
	Employed	5 (0.3)	745 (50.1)	686 (46.2)	50 (3.4)		
Income	Less than 100$	1 (0.2)	236 (57)	168 (40.6)	9 (2.2)	22.16	0.001
	100-300$	3 (0.3)	428 (49.7)	405 (47)	25 (2.9)		
	More than 300$	1 (0.4)	104 (41.1)	132 (52.2)	16 (6.3)		
Fertility Status	Fertile	3 (0.3)	510 (50.1)	467 (45.9)	37 (3.6)	1.38	0.71
	Infertile	2 (0.4)	258 (50.5)	238 (46.6)	13 (2.5)		

**Table 4:** the relation between socio-demographic variables and idealistic distortion levels

**Variable**		**Idealistic Distortion**				**χ** ^2^	**p Value**
		**Low** **n (%)**	**Average** **n (%)**	**High** **n (%)**	**Very High** **n (%)**		
Place of Residence	Urban	3 (0.5)	189 (29.3)	318 (49.3)	135 (20.9)	5.314	0.105
	Rural	13 (1.5)	243 (27.5)	420 (47.6)	207 (23.4)		
Job	Employed	4 (1.8)	63 (27.9)	113 (50)	46 (20.4)	1.98	0.58
	Housewife	12 (0.9)	369 (28.3)	625 (48)	296 (22.7)		
Education	Illiterate	1 (2.6)	7 (18.4)	24 (63.2)	6 (15.8)	10.55	0.31
	Below diploma	10 (1.4)	212 (29.7)	332 (46.6)	159 (22.3)		
	High school Diploma	2 (0.4)	141 (29.1)	232 (47.9)	109 (22.5)		
	Higher Education	3 (1)	72 (24.6)	150 (51.2)	68 (23.2)		
Spouse’s Education	Illiterate	0 (0)	0 (0)	4 (80)	1 (20)	11.99	0.214
	Below diploma	11 (1.5)	220 (29.7)	334 (45.1)	175 (23.6)		
	High school Diploma	1 (0.6)	54 (31)	87 (50)	32 (18.4)		
	Higher Education	4 (0.7)	158 (25.9)	313 (51.4)	134 (22)		
Spouse’s Job	Unemployed	2 (4.8)	18 (42.9)	18 (42.9)	4 (9.5)	12.40	0.006
	Employed	14 (0.9)	414 (27.9)	720 (48.5)	338 (27.7)		
Income	Less than 100$	7 (1.7)	105 (25.4)	197 (47.6)	105 (25.4)	12.68	0.048
	100-300$	7 (0.8)	237 (27.5)	431 (50.1)	186 (21.6)		
	More than 300$	2 (0.8)	90 (35.6)	110 (43.5)	51 (20.2)		
Fertility Status	Fertile	10 (1)	288 (28.3)	503 (49.5)	216 (21.2)	2.75	0.433
	Infertile	6 (1.2)	144 (28.2)	235 (46)	126 (24.7)		

## Discussion

In our study the mean percentage scores and level of marital satisfaction in fertile and infertile women showed no significant difference which is in line with the findings of Amanelahifard and colleagues (25) and Ferreira and colleagues (26). In a study entitled “The comparison of marital satisfaction between fertile and infertile women” reported a significant difference in the levels of marital satisfaction in fertile and infertile women in Tehran which is not consistent with the current results (21). Onat and colleagues (22) in a study entitled “Marital relationship and quality of life among couples with infertility” emphasized the impact of infertility on marital relationships which is not consistent with the current results either. Valsangkar and et.al pointed to the impact of infertility on marital satisfaction, which is not consistent with recent results (8). Monga and colleagues also demonstrated that marital adjustment in infertile couples was significantly less than that in fertile group which is not consistent with the present results (4). Onat and colleagues in a study entitled “The Effects of infertility on gender differences in marital relationship and quality of life: A case-control study of Turkish couples” pointed to the lack of difference between fertile and infertile groups which is consistent with the current results (27).

In a study entitled “Relation between psychosocial factors and marital satisfaction in infertile women” showed that infertile participants had low marital satisfaction which is inconsistent with the present results (17). Jonaidy and colleagues’ study in Mashhad (East of Iran) indicated higher marital satisfaction in infertile women, which is not consistent with recent results (20). Hatamloye and et.al reported no significant differences in marital satisfaction in fertile and infertile women (28). Perhaps it can be claimed that the considerable advances in the treatment of infertility and assisted reproductive technology have boosted hope in the couples and has caused less trouble in marital relations among infertile couples and stages of infertility treatment have divided the stress between the spouses and have strengthened the marital relationship and intimacy between them.

In this study also significant relationships were found between marital satisfaction, job, spouses’ jobs, and family income. But no significant association was observed between marital satisfaction and the place of residence, fertility status, age at marriage and marriage duration, which is consistent with the results of Jonaidy (20). Heidari and et al. pointed to lack of relationship between duration of marriage and marital satisfaction, which is consistent with the recent study (17). Valsangkar and et al. in their study noted a relationship between age, economic status and fertility status, which is consistent with part of the present results (8). Onat and colleagues in their study found no association between education and occupation, and marital satisfaction in both fertile and infertile women, and they also showed a relationship between age and marital satisfaction in both groups, which is consistent with part of recent results (22).

Ferreira and colleagues in a study in Portugal entitled “Influence of infertility and fertility adjustment on marital satisfaction” reported a relationship between marital satisfaction with the level of education and age of the infertility treatment onset which is consistent with part of the recent results (26). 

The current results also showed no significant association between marital satisfaction and fertility status, which is consistent with Ferreira and colleagues’ results indicating lack of significant differences between fertile and infertile women (26). In a another study, the researchers pointed to the lower mean scores on this dimension in infertile women and reported a significant difference between the two groups, which is not consistent with the present results (3). 

The present study (Table 4) also showed a significant relationship between idealistic distortion and spouse’s education and income, but no significant differences were observed between the two groups in place of residence, job, education, spouse’s education and fertility status. Katiraei and et al. in their study referred to the lower scores of infertile women on this dimension and a significant difference was noted between the two groups, which is not consistent with the present results (3). Lower scores of infertile women on this dimension indicate that they are realistic about marital relationships.

 Women participation in this study is one of the limitations of the study. Since the issue of marital satisfaction is among the most private issues of individuals and due to the cultural and religious limitations of society in Iran, people cannot easily express their marital problems. Therefore, the probability of not stating the facts about marital problems is another limitation of the study which was out of the researchers’ control. Another limitation of the study is the lack of studies on the subscales of the present study which has impoverished the discussion of the findings.

Results showed that fertility status does not decrease marital satisfaction. Since marital satisfaction is moderate in both groups, sex education in schools and universities and for marrying people and sexual counseling for the couples can lead to improved sexual satisfaction. Also high rates of idealistic distortion in both groups indicate unrealistic expectations of sex relationships which are based on acceptable social behaviors. Therefore, midwifery and health care consultations can help to make the expectations of marital relationships more realistic.

## References

[1] Pazandeh F, Sharghi S, Karami-Nouri R, Alavi H (2005). A comparative study of psycho-social aspects between infertile and fertile women referring to a health cares center and infertility center in Tehran/2003. Pejouhandeh.

[2] Akhondy M, Sadeghy M (2003). Psychological impact of infertility. IJRM.

[3] Katiraei S, Haghighat M, Bazmi N, Ramezanzadeh F, Bahrami H (2010). The Study of Irrational Beliefs, Defense Mechanisms and Marital Satisfaction in Fertile and Infertile women. Journal of Family and Reproductive Health.

[4] Monga M, Alexandrescu B, Katz SE, Stein M, Ganiats T (2004). Impact of infertility on quality of life, marital adjustment, and sexual function. Urology.

[5] Rascanu R, Vladica S (2012). Attitudinal and emotional structures specific for infertile women. Procedia-Social and Behavioral Sciences.

[6] Marci R, Graziano A, Piva I, Lo Monte G, Soave I, Giugliano E (2012). Procreative sex in infertile couples: the decay of pleasure?. Health Qual Life Outcomes.

[7] Shindel AW, Nelson CJ, Naughton CK, Ohebshalom M, Mulhall JP (2008). Sexual function and quality of life in the male partner of infertile couples: prevalence and correlates of dysfunction. The Journal of Urology.

[8] Valsangkar S, Bodhare T, Bele S, Sai S (2011). An evaluation of the effect of infertility on marital, sexual satisfaction indices and health-related quality of life in women. Journal of Human Reproductive Sciences.

[9] Oskay UY, Beji NK, Serdaroglu H (2010). The issue of infertility and sexual function in Turkish women. Sexuality and Disability.

[10] Zegers-Hochschild F, Adamson GD, de Mouzon J, Ishihara O, Mansour R, Nygren K (2009). international committee for monitoring assisted reproductive technology (ICMART) and the world health organization (WHO) revised glossary on ART terminology, 2009. Human Reproduction.

[11] Balen A (2008). Infertility in practice.

[12] Turan V, Kopuz A, Ozcan A, Kocakaya B, Sahin C, Solmaz U (2014). Sexual dysfunction in infertile Turkish females: prevalence and risk factors. Eur J Obstet Gynecol Reprod Biol.

[13] Zargar AH, Wani AI, Masoodi SR, Laway BA, Salahuddin M (1997). Epidemiologic and etiologic aspects of primary infertility in the Kashmir region of India. Fertil Steril.

[14] Kumar D (2007). Prevalence of female infertility and its socio-economic factors in tribal communities of Central India. Rural and Remote Health.

[15] Benson RC (1983). Handbook of Obstetrics & Gynecology.

[16] Turan V, Oktay K (2014). Sexual and fertility adverse effects associated with chemotherapy treatment in women. Expert Opin Drug Saf.

[17] Heidari P, Latifnejad R (2010). Relationship between psychosocial factors and marital satisfaction in infertile women. The Journal of Qazvin Univiversity of Medical Sciences.

[18] Hammarberg K, Astbury J, Baker H (2001). Women's experience of IVF: a follow-up study. Human Reproduction.

[19] Salehy Fadardy J (1999). The development and validation of marital satisfaction questionnaire on a sample of students of Ferdowsi university. Psychother Novelties.

[20] Jonaidy E, Sadodin S, Mokhber N, Shakeri M (2009). Comparing the marital satisfaction in infertile and fertile women referred to the public clinics in Mashhad in 2006-07. Iranian Journal of Obstetrics, Gynecology and Infertility.

[21] Bahrainian SA, Nazemi F, Dadkhah A (2009). The comparison of marital satisfaction between fertile and infertile women. Iranian Rehabilitation Journal.

[22] Onat G, Beji NK (2012). Marital relationship and quality of life among couples with infertility. Sexuality and Disability.

[23] Fowers BJ, Olson DH (1989). ENRICH Marital Inventory: A discriminant validity and cross-validation assessment. Journal of Marital and Family Therapy.

[24] Asoodeh MH, Khalili S, Daneshpour M, Lavasani MG (2010). Factors of successful marriage: Accounts from self described happy couples. Procedia-Social and Behavioral Sciences.

[25] Amanelahifard A, Nikbakht R, Hoseini MA, Fakhr SA, Hoseini Z (2012). The comparison of marital satisfaction and quality of life between fertile and infertile women. Biannual Journal of Applied Counseling.

[26] Ferreira M, Antunes L, Duarte J, Chaves C (2015). Influence of infertility and fertility adjustment on marital satisfaction. Procedia-Social and Behavioral Sciences.

[27] Onat G, Beji NK (2012). Effects of infertility on gender differences in marital relationship and quality of life: a case-control study of Turkish couples. Eur J Obstet Gynecol Reprod Biol.

[28] Hatamloye Saedabadi M, Hashemi Nosratabad T (2012). The comparison of psychological well-being and marital satisfaction in the fertile and infertile women. Health Psychology.

